# A scoping review of tinnitus research undertaken by New Zealand researchers: Aotearoa–an international hotspot for tinnitus innovation and collaboration

**DOI:** 10.1080/03036758.2024.2363424

**Published:** 2024-07-03

**Authors:** Grant Searchfield, Divya Adhia, Amit Barde, Dirk De Ridder, Maryam Doborjeh, Zohreh Doborjeh, Ronald Goodey, Michael R. D. Maslin, Phil Sanders, Paul F. Smith, Yiwen Zheng

**Affiliations:** a School of Population Health, Faculty of Medical and Health Sciences, The University of Auckland, New Zealand; b Eisdell Moore Centre, School of Population Health, Faculty of Medical and Health Sciences, The University of Auckland, New Zealand; c TrueSilence Therapeutics Inc, Atlanta, Georgia, USA; d Section of Neurosurgery, Department of Surgical Sciences, Dunedin School of Medicine, University of Otago, Dunedin, New Zealand; e Knowledge Engineering and Discovery Research Institute, School of Engineering, Computer and Mathematical Sciences, Auckland University of Technology, Auckland, New Zealand; f School of Psychology, Speech and Hearing, The University of Canterbury, Canterbury, New Zealand; g Dept. of Pharmacology and Toxicology, School of Biomedical Sciences, University of Otago, Dunedin, New Zealand

**Keywords:** Innovation, Mechanisms, New Zealand, Therapy, Tinnitus, Treatment

## Abstract

Tinnitus is a very common oto-neurological disorder of the perception of sound when no sound is present. To improve understanding of the scope, strengths and weaknesses of New Zealand tinnitus research, a critical scoping review was undertaken. The aim was to help develop priorities for future research. A review of the literature was undertaken using a 6-stage scoping review framework of Scopus and Pub Med were searched in May 2023 with the combination of following key word [Tinnitus] and country of affiliation [New Zealand]. The search of PubMed resulted in 198 articles and that of Scopus 337 articles. After initial consideration of title relevance to the study (165 from PubMed and 196 from Scopus) removal of duplicates and after reading the articles and adding from references, 208 studies were chosen for charting of data. Nine themes were identified and described: A. Epidemiology; B. Models; C. Studies in animals; D. Mechanisms; E. Assessment and prognosis; F. Pharmacotherapy; G. Neuromodulation; H. Sensory therapies; I. Clinical practice. An urgent priority for future tinnitus research in NZ must be to address the absence of cultural and ethnic diversity in participants and consideration of traditional knowledge.

## Introduction

This scoping review was undertaken to understand the contribution of Aotearoa/New Zealand (NZ) research to understanding tinnitus, its assessment and management. Tinnitus (Tī ngā taringa in Te Reo Māori), commonly known as ‘ringing in the ears’, is a phantom perception created by brain activity usually in response to head or ear injury. The condition is very common, with prevalence of approximately 10% in the United States (Bhatt et al. 2016). Tinnitus is very heterogeneous in its presentation, cause and underpinning neurophysiology. There are multiple classification schemes for tinnitus according to its origin, duration and impact. Subjective tinnitus is true tinnitus, that is, a sound perceived in the absence of a corresponding sound source. Objective tinnitus (also called somatosounds) are true sounds created by the body, for example, pulsatile sounds due to audible blood flow or clicking sounds coming from the soft palate. Objective tinnitus may be heard by other people–possibly made audible by listening with a stethoscope. This scoping review excludes objective tinnitus. Tinnitus can be a brief experience, for example ‘nightclub’ tinnitus, or can last many years. The crossover between acute and chronic tinnitus is vague, but typically is considered chronic after three to six months of onset (De Ridder, Schlee, et al. 2021). The impact of tinnitus can vary from slight annoyance through to a negative impact on daily activities. This has led to the differentiation between ‘tinnitus’, being hearing the tinnitus sound, and ‘tinnitus disorder’, which is associated with suffering and poor quality of life; disrupted hearing, impaired attention, reduced sleep and potentially anxiety and depression (De Ridder, Schlee, et al. 2021).

Currently no single treatment can eliminate the perception of tinnitus. Reducing its impact is possible but this is complicated by its heterogeneity (Mohan et al. 2022). The complexity of tinnitus has resulted in multidisciplinary international collaborative efforts to discover its mechanisms and improve clinical outcomes. New Zealand researchers have been significant contributors to these international efforts. New Zealand has been well represented in international tinnitus scientific advisory boards and organisations (e.g. Tinnitus Australia, American Tinnitus Association, Tinnitus Research Initiative), and researchers have contributed to international research standards and consensus policies. New Zealand hosted the major annual tinnitus conference, the Tinnitus Research Initiative in 2014. Bibliometric analyses of the tinnitus literature identified NZ researchers and NZ Universities as being important contributors to international tinnitus research networks (Zhou et al. 2022; Ye et al. 2023). Expertscape (https://expertscape.com/ex/tinnitus) ranks NZ in the top 10 countries internationally for tinnitus publications (without any per capita correction).

The aim of this scoping review was to summarise all the material available about tinnitus research in NZ. Documentation of research within NZ and collaborations external to NZ will illustrate the tinnitus research landscape to identify NZ tinnitus research strengths and weaknesses. It will aid in the development of strategic plans to fill the gaps in our understanding and develop priorities for future research funding. Ultimately this knowledge will be used to improve the well-being of persons with tinnitus, not only in NZ, but globally. The scoping approach is particularly useful with such a complex, heterogeneous disorder and the multidisciplinary nature of the research.

## Methods

**Figure 1.**
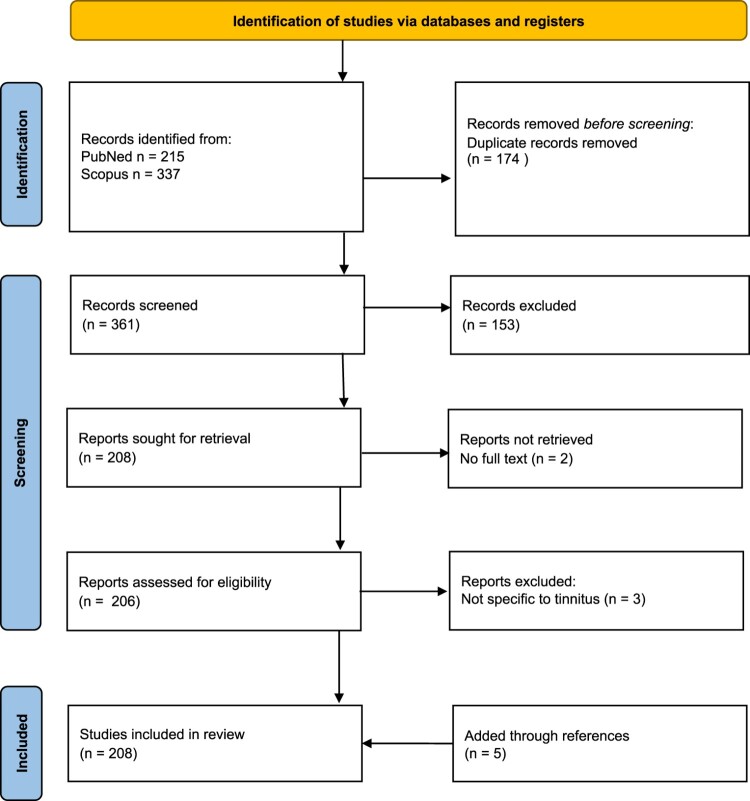
PRISMA 2020 flow diagram.

A review of the literature was undertaken using a six-stage scoping review framework (Arksey and O’Malley 2005). PRISMA guidelines were followed (Figure 1; Page et al. 2021).

Stage 1. Identifying the research question.

The research question was ‘what is NZ’s contribution to research on tinnitus, its assessment and management?’.

Stage 2. Identifying relevant studies.

To identify relevant studies, an intensive search was carried out in May 2023 (by author GDS) using the databases Scopus and PubMed with the combination of the following key word [Tinnitus] and country of affiliation [New Zealand]. Reference lists of the articles were reviewed and hand searching of key journals about the topic was undertaken. The search of PubMed resulted in 215 articles and that of Scopus 337 articles. After initial consideration of title relevance to the study (165 from PubMed and 196 from Scopus) and removal of duplicates, 206 articles were shortlisted, then, after reading the articles and checking references, 208 studies were chosen for charting of data.

Stage 3. Study selection.

Articles were excluded if: tinnitus was not central to the research question (e.g. research of Meniere’s disease with tinnitus as a symptom), the tinnitus was of the objective type (somatosound) or the full text was unavailable. Research undertaken outside of NZ by NZ researchers was included; the nature of the research and participant population was described in a commentary accompanying the citation. Unpublished student theses were excluded from selection. Published abstracts without the full text were excluded. Conference proceedings available in full-text form were included.

Stage 4. Charting the data.

Initial arrangement of the publications into themes was undertaken (by GDS) based on the title and abstract. This initial organisation of the data was then evaluated and agreed upon by all co-authors. Nine broad themes were identified: A. Epidemiology; B. Models; C. Studies in animals; D. Mechanisms; E. Assessment and prognosis; F. Pharmacotherapy; G. Neuromodulation; H. Sensory therapies; I. Clinical practice. Subject matter experts reviewed the compiled articles and recommended inclusion or exclusion based on full text review, and summarised the themed data as a narrative review. This necessitated some self-citation. To reduce bias in selection and commentary, each section was reviewed by all authors.

Stage 5. Collating the results.

The narrative for each theme was assembled along with summary statistics (number of articles, year of publication range). As this was a scoping review, and given the diversity in methods and research disciplines, a meta-analysis was not undertaken. Sample sizes were recorded. When the results were obtained with a NZ participant population specific commentary was made in the discussion on the implications for NZ tinnitus sufferers and/or NZ tinnitus health delivery.

Stage 6. Consultation.

Research stakeholders were consulted as to their interpretation of the results. Following this, the results were discussed with a focus on answering the research question and identifying strengths and gaps in NZ tinnitus research.

## Results

### Epidemiology

**Figure 2.**
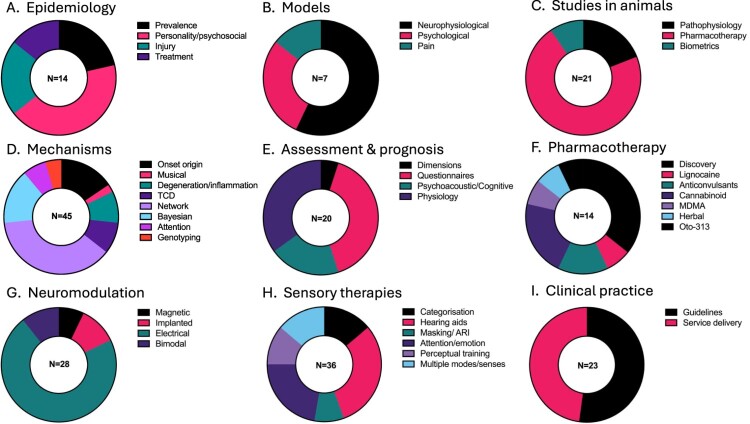
Proportion of articles within each theme. **A,** Epidemiology; **B,** Models; **C,** Studies in animals; **D,** Mechanisms; **E,** Assessment and prognosis; **F,** Pharmacotherapy; **G,** Neuromodulation; **H,** Sensory therapies; **I,** Clinical practice. TCD–Thalamocortical Dysrhythmia, OTO-313–a sustained-exposure formulation of gacyclidine, ARI–acoustic residual inhibition.

Fourteen articles published between 1989–2021 were reviewed (Figure 2A). The overall weighted prevalence for tinnitus in the NZ population was 6% (sample *n* = 69,976) (Wu et al. 2015). The prevalence was higher in males (6.5%) compared to females (5.5%) and increased with age (13.5% aged 65 and over). Amongst NZers <65 years old, persons identifying as European were more likely to report tinnitus (5.48%) than Māori (2.41%), Pasifika (0.89%), Asian (0.92%) and ‘Other’ ethnicity (4.39%). The prevalence amongst older NZers (age 65 or older) was higher. In this age group prevlaence was similar among the different ethnic groups (European, 13.69%, Māori 11.23%, Pasifika 10.39%, Other 13.47%) except for Asian peoples who reported tinnitus less (5.80%). The Dunedin Multidisciplinary Health and Development study (‘Dunedin study’) has offered insight into various predictors of tinnitus based on regular testing of an initial sample of 1037 participants born in 1972–1973. At 32 years of age, participants (*n* = 970) were asked if they had experienced tinnitus in the preceding 12 months; 45% had, with 6.8% reporting ‘half the time or more’ (Dawes and Welch 2010). Complicating interpretation of epidemiological studies is variation in tinnitus definitions. Several NZ researchers have contributed to this debate, resulting in the proposal of distinguishing between ‘tinnitus’ and ‘tinnitus disorder’ (De Ridder, Schlee, et al. 2021). Tinnitus disorder is when tinnitus is associated with emotional distress, cognitive dysfunction and/or autonomic arousal, leading to behavioural changes and functional disability. A scoping review of 60 international studies identified personality traits as being consistently associated with tinnitus help-seeking (Durai and Searchfield 2016). This was confirmed in a NZ sample; higher levels of stress reaction and alienation, lower social closeness and lower self-control were found in a chronic tinnitus group (*n* = 154) compared to a control group (*n* = 61) (Durai et al. 2017). The Dunedin study identified that 32-year-olds with tinnitus were more socially withdrawn, reactive to stress, alienated and had less self-control (Welch and Dawes 2008). Welch and Dawes (2008) interpreted that personality determined a predisposition to interpret a signal as being present. Tinnitus might comprise 3 components: 1. Ignition; 2. Promotion resulting in cortical representation of the tinnitus signal; and 3. Awareness based on the theory of signal detection (Welch and Dawes 2010). Personality may also be an important factor in treatment selection. Conscientiousness and openness predicted therapy benefit in a German-language Internet Cognitive Behavioural Therapy (Kleinstäuber et al. 2018).

Many factors can cause or aggravate tinnitus, for example, loud noise exposure induced tinnitus in 49 of 53 participants (George and Kemp 1989). There have been fears that the more regular exposure to loud music made possible with digital music platforms could result in higher numbers of youths with permanent hearing loss and tinnitus. A survey of high school students in Belgium found that temporary tinnitus was reported by 75% of students (Gilles et al. 2013). Hearing damage is not the only determining factor for tinnitus; trauma, personality and social factors also play significant roles. Over 50% of individuals with traumatic brain injury develop tinnitus, but there is limited research on the topic (Kreuzer et al. 2014). The transition from an acute tinnitus experience to chronic tinnitus, potentially tinnitus disorder, may be determined by psychosocial factors (Kleinstäuber and Weise 2021). Kleinstäuber and Weise (2021) undertook a systematic review and identified that distress, somatic complaints, delayed sleep onset, active illness, as well as personality, predict transition from acute to chronic tinnitus. Addressing these factors early may reduce the risk of temporary tinnitus becoming chronic. A key factor in this is access to appropriate clinical help (Kleinstäuber and Weise 2021). There is no recent national survey of treatment effectiveness in NZ. However, in a sample of NZ tinnitus sufferers (*n* = 336) 30 years ago, 85% reported seeking help with tinnitus; of these, 49% were offered ineffective treatments, and for 10% their tinnitus was reduced or eliminated (George and Kemp 1991). Lifestyle changes modified severity, according to diaries by nine participants followed for three months (Kemp and George 1992a).

### Models

Seven articles between 2010–2022 were reviewed (Figure 2B). Tinnitus models are theories derived from empirical research to help explain the relationship between observations and experimental data relating to the onset, maintenance and treatment of tinnitus. As understanding of tinnitus mechanisms has increased, models reflecting the complexity of interplay between the auditory system, the brain regions associated with attention, memory and emotion, and the environment have developed. These tinnitus models share common themes with models of chronic or phantom pain (Magnusson 2010). Most models in tinnitus are neurophysiological (De Ridder, Vanneste, Weisz, et al. 2014; De Ridder, Vanneste, Langguth, et al. 2015; De Ridder and Vanneste 2021; De Ridder et al. 2022) based on measurements either in animals (see animals studies section) or humans (mechanisms section), but may also be based on psychological principles (Searchfield et al. 2012; Searchfield, 2014).

From an ecological psychoacoustics perspective, tinnitus can be considered the perception of an auditory event that cannot be validated within the context of environment, relationships and beliefs as well as personality and memories of true sound and silence (Searchfield 2014). In this model tinnitus is incongruent with learnt, predictable patterns both in terms of how sound behaves and its meaning. The loudness of tinnitus in this model is based on ‘Adaptation Level Theory’; how tinnitus magnitude varies according to prior experiences, context, its ability to capture attention and individual susceptibility to negative reactions (Searchfield et al. 2012).

**Figure 3.**
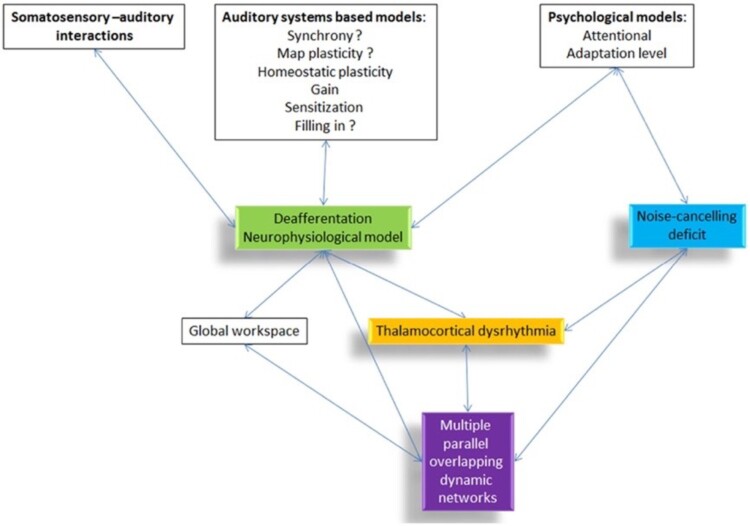
Overview of different tinnitus models and how they are related to each other (De Ridder, Vanneste, Langguth, et al. 2015).

Physiological models explain how the psychological observations occur and cast light on potential anatomical and functional targets for therapeutics. The different dimensions of tinnitus have been modelled as a cascade of events following deafferentation or failed neural noise cancelling (De Ridder, Vanneste, Langguth, et al. 2015) that results in changed connectivity and activation of multiple parallel networks (De Ridder, Vanneste, Weisz, et al. 2014). Variance in activity within these networks can help explain the heterogeneity in tinnitus experience. Recently the model has been refined as a ‘Triple Network Model’ in which chronic tinnitus can be separated functionally and anatomically into three pathways consisting of a lateral pathway coding the tinnitus ‘sound’, a medial ‘suffering’ pathway and a descending ‘noise-cancelling’ pathway (De Ridder et al. 2022). The ‘Bayesian Brain’ model of tinnitus (De Ridder and Vanneste 2021) posits that tinnitus is a new reference state created by an imbalance between the lateral and medial pathways. Like the psychological ecological model of tinnitus, the Bayesian brain model captures the need for the brain to make predictions and then respond appropriately, including learning from ‘prediction errors’. Upon deafferentation, if precision (synaptic gain) rises, tinnitus is perceived, with attention to the signal further increasing precision; with time this chronic activity becomes the new reference state. The Bayesian model supports conditioning as a therapy. Positive reinforcement behaviourally (using reward of non-tinnitus sound perception and not rewarding tinnitus-matched sounds) or through direct electrical stimulation should reduce tinnitus salience (De Ridder and Vanneste 2021). Thalamocortical dysrhythmia (TCD) may be a common mechanism connecting the psychological and neurophysiological network models (Figure 3). TCD is a change in thalamocortical signal transmission thought to reflect errors in predicted versus sensed auditory input (De Ridder, Vanneste, Langguth, et al. 2015). The NZ research on mechanisms underpinning these models will be described in later sections.

### Studies in animals

Twenty-one articles between 2004–2021 were reviewed (Figure 2C). Animal studies enable researchers to deliver, control and measure the outcomes of specific interventions at a cellular level, not possible in humans with pre-existing tinnitus. Translating findings from animal studies to humans and vice-versa has been challenging in tinnitus research. In the past, the presence and characteristics of tinnitus in animals have had to be assumed, on the basis of similar pathophysiology to humans (Searchfield et al. 2004). This limitation was reduced with the development of behavioural conditioning paradigms that demonstrate animals hear tinnitus. After exposure to tinnitus inducing pathophysiology through exposure to loud noise, or high doses of sodium salicylate, animals behave in silence as if sound was present. Tinnitus also has a stress response and it affects both cognition and sleep in humans. In animals with tinnitus following acoustic trauma, behaviour changed, for example they became more aggressive, but there were no significant changes in anxiety compared to control animals (Zheng, Hamilton, McNamara, et al. 2011). In another group of animals, acoustic trauma-induced tinnitus impaired impulse control, but not accuracy at an attentional task (Zheng, Hamilton, Stiles, et al. 2011). Acute stress-induced sleep disturbance did not exacerbate tinnitus perception in rats (Zheng, Stiles, et al. 2014).

No drug has been approved by the United States Food and Drug Administration for Tinnitus (Zheng et al. 2021). Smith and Zheng (2021) propose a data-driven approach to drug discovery using multivariate data mining techniques to uncover hidden patterns and relationships (Smith and Zheng 2021). This exploration holds the potential for identifying biomarkers in animals, and then in humans, for diagnosis and deeper insights into the interconnected nature of tinnitus mechanisms. Researchers at Otago University have been able to investigate multiple putative tinnitus drugs using a conditioned behavioural suppression paradigm. These include the testing of drugs with either GABAergic enhancing properties (Zheng, Vagal, et al. 2012) or glutamatergic reducing properties (Zheng, McNamara, et al. 2012). L-baclofen (a GABA_B_ receptor agonist) dose-dependently reduced the behavioural signs of chronic tinnitus in an animal model caused by acoustic trauma (Smith et al. 2012; Zheng, Vagal, et al. 2012), but failed to prevent tinnitus post-acoustic trauma and did not alter GABA receptor expression in the cochlear nucleus according to another study (Zheng, McPherson, et al. 2014). A decrease in GABAergic inhibition at the cochlear nucleus may not be responsible for tinnitus, as noise exposure associated with a tinnitus-indicative behavioural change was not associated with glutamic acid decarboxylase and enzymes responsible for GABA synthesis (Zheng, Dixon, et al. 2015). Blocking glutamate receptors appears more promising (also see pharmacotherapy in humans section). The N-methyl-D-aspartate (NMDA) glutamate receptor antagonist memantine reduced the number of noise-exposed animals demonstrating tinnitus behaviours (Zheng, McNamara, et al. 2012). Following salicylate, the anti-epileptic carbamazepine, which reduces glutamate in the brain, also has been demonstrated to suppress tinnitus (Zheng et al. 2008).

Drugs that modulate other neurotransmitter systems, such as the cholinergic, serotoninergic, noradrenergic and endocannabinoid systems, have also been tested in animal models of tinnitus (Zheng et al. 2021). The activation of the endocannabinoid system may promote the development of tinnitus (Zheng, Stiles, et al. 2010; Zheng et al. 2015). Down regulation of cannabinoid CB_1_ receptors has been identified in the ventral cochlear nucleus (VCN) following salicylate (Zheng et al. 2007). Increased neuronal excitability may occur from nitric oxide neurotransmitter release. Neurons in the VCN increase expression of neuronal nitric oxide synthase, a precursor to nitric oxide, following salicylate treatment (Zheng et al. 2006).

There has recently been interest in neuroinflammation as a mechanism for tinnitus. Melanocortins, neuropeptide hormones, have promise therapeutically in neuroprotection and inflammation. In an acoustic-trauma animal model of tinnitus, a melanocortin agonist did not protect against tinnitus (Zheng, McPherson, et al. 2015). Traditional Chinese medicine has been applied to tinnitus, but at least one combination, ‘Er Ming Fang’, did not have any significant effect on salicylate-induced tinnitus in rats (Zheng, Vagal, et al. 2010).

Many candidate drugs prove ineffective when tested in animals. Developing personalised treatment is seen as the future of tinnitus therapy. In this regard, animal studies have indicated the potential for large-scale metabolic profiling methods to identify biomarkers and subtype tinnitus patients (He et al. 2017, 2021).

### Mechanisms in humans

Forty-five articles were included from 1986–2023 (Figure 2D). Tinnitus can be acute following short duration intense sound exposure (Kemp and Plaisted 1986) or chronic, most commonly following cochlear pathophysiology (Gilles, De Ridder, et al. 2013), such as occurs with longer noise exposure (Thorne 2010). Peripheral inputs, including from the vestibular system (Smith 2012), may modulate tinnitus via the dorsal cochlear nucleus, but over time it is central reorganisation as a stabilising response to deafferentation that may create the perception of sound (Mohan et al. 2016). After the initial injury, correlates of tinnitus perception and disorder are reflected in central nervous system activity, quantified using neuroimaging (Langguth, Elgoyhen, et al. 2013; Elgoyhen et al. 2015). Tinnitus is usually a simple sound (tonal, buzzing, noise-like) but music is sometimes also heard. Musical hallucinations are much less common than typical tinnitus, and differ from typical tinnitus in being associated with memories of real music and the activation of music and language processing regions of the brain (Vanneste, Song, et al. 2013).

Altered functional connectivity associated with tinnitus has been investigated with resting-state EEG and fMRI. Functional measures appear more effective in studying tinnitus than voxel-based morphometry identifying anatomical changes (Vanneste et al. 2015). Hyperexcitable activity can lead to altered synchrony of activity between neurons or neural populations, with those disruptions in synchrony potentially leading either to an increase or a decrease in functional or anatomical connectivity within or between brain regions (Song et al. 2017; Knipper et al. 2020). Associated tinnitus mechanisms might include metabolic changes like neuroinflammation (Shulman et al. 2021) and developmental (early onset) or neurodegenerative (late onset) age-related effects (Song, De Ridder, et al. 2013; Yoo et al. 2016a, 2016b). Tinnitus has been characterised to minimally consist of two networks, one that encodes loudness and another distress (Vanneste, Congedo, et al. 2014). But it appears that there are multiple spatiotemporally overlapping networks that reflect additional tinnitus properties depending on the degree of disorder experienced (Mohan et al. 2017). An increase in functional linkage between the auditory cortex and frontal and parahippocampal areas appear to be prerequisites for tinnitus generation (Vanneste, Joos, et al. 2014; Vanneste et al. 2018; Vanneste, To, et al. 2019). Tinnitus disorder demonstrates altered connectivity within the so-called salience (or distress) network involving cingulate cortices, parrahippocampal regions and large portions of the limbic system (Mohan et al. 2018, 2020; Hullfish et al. 2018b; Lee et al. 2021). Tinnitus awareness, loudness and distress are correlated but distinct tinnitus properties. Tinnitus awareness was negatively correlated with the rostral anterior cingulate cortices. In one study, the loudness of tinnitus involved the nucleus accumbens and parahippocampal cortex (Hullfish et al. 2019); in another, subjective loudness ratings were correlated with the anterior cingulate/insula, the parahippocampus and auditory cortex activity, while tinnitus matched to the intensity of a sound did not correlate with any EEG measures (De Ridder, Congedo, et al. 2015). The perceived loudness of tinnitus, obtained using ratings, is known to differ from matching to external sounds (psychoacoustical matching). A person may say their tinnitus is very loud but match it to a low sensational level of sound. An increase in temporal variability in resting-state EEG occurs with tinnitus and increases with increasing tinnitus loudness, not distress, suggesting tinnitus distress may be more hardwired (Mohan et al. 2018). However, persons with tinnitus and panic disorder do have reduced connectivity between the dorsal anterior cingulate cortex and the amygdala, insula and precuneus compared to those without panic disorder (Pattyn et al. 2018). The interaction between these networks is complex and comprises correlations and anti-correlations that depend on the levels of distress (Vanneste and De Ridder 2015). There have been efforts at developing therapeutic strategies based on influencing the connectivity between target regions. For example, Vanneste et al. (2015) studied patients who, via the Tinnitus Questionnaire, were categorised as experiencing clinically significant distress. Brain activity was characterised before and after a period of neurofeedback training, whereby brain rhythms in different scalp regions were displayed to the individual as a visual pattern of lights. The individual was asked to focus their attention on changing the visual pattern (e.g. increase the height of a light on a screen), so that in this way their attention controls the visual display, and the individual can learn via feedback. However, the source of the brain rhythms can be modified so that the brain rhythms might correspond to a brain region known to be relevant to tinnitus generation or distress, or a control region involving a brain region not associated with tinnitus. Connectivity analysis before and after a period of training showed that, when training involved brain regions involved in tinnitus distress, like the posterior cingulate cortex, the amount of distress was reduced after training. On the other hand, when training involved brain regions not related to tinnitus distress, like the lingual gyrus, no effect was observed in either distress or functional connectivity.

The Bayesian brain model expresses the view that a key feature of sensory processing and, in particular, top-down modulation of sensory input, is to predict auditory events based on existing memory representations, with the ensuing benefit of reducing uncertainty around the content of impoverished input but also to minimise the signal processing burden (Durai, O’Keeffe, et al. 2018a). Auditory-evoked potential indices of streaming and prediction were found to differ between tinnitus (*n* = 17) and controls (*n* = 14) (Durai et al. 2019). The absence of tinnitus amongst the congenitally deaf suggests the experience of processing sound, possibly to develop auditory predictions, is a prerequisite for tinnitus generation (Lee et al. 2017; Knipper et al. 2020). The Bayesian brain model predicts that different tinnitus-generating mechanisms should exist based on the amount of hearing loss, i.e. how much modulation and inference from existing memory might be necessary (De Ridder, Joos, et al. 2014; Song et al. 2021). With relatively little hearing loss, less inference should be necessary. The experience of tinnitus, with normal pure-tone thresholds, could be related to different amounts of Bayesian inference. Vanneste and De Ridder (2016) investigated this hypothesis by comparing the data from individuals with tinnitus and little or no hearing loss with that from individuals with tinnitus and a clinically significant hearing loss, along with a third group of healthy controls. The data primarily consisted of resting-state brain rhythms obtained via EEG, with alterations in the alpha brain waves but also lower (theta) and higher (gamma) frequency responses in the tinnitus group compared with controls. The individuals with tinnitus and minimal hearing loss only showed a correlation between the subjective loudness of their tinnitus and the brain rhythms localised to the auditory cortical areas. On the other hand, the individuals with tinnitus and a measurable hearing loss showed the opposite pattern with a stronger correlation between the loudness of their tinnitus and activity localised to the parahippocampal areas (Vanneste and De Ridder 2016).

Thalamocortical dysrhythmia (TCD) is one model used to explain observable characteristics of altered functional connectivity within or between brain regions (Vanneste, Alsalman, et al. 2019; To et al. 2021). With TCD, we expect the normally observed alpha (8 Hz–12 Hz) brain waves to be reduced in magnitude with a relative increase in both lower (theta) and higher (gamma) frequency brain waves, particularly associated with temporal regions of the brain where the auditory cortices reside. Lee et al. (2020) compared the resting EEG data with sudden sensorineural hearing loss (SSNHL) and associated tinnitus, and with SSNHL of a similar severity and pattern, but without tinnitus. The results showed increased brain activity, particularly in the gamma frequency range, in the tinnitus group along with an associated increase in the functional connectivity between frontal cortical regions associated with emotional responses to sensation. In another example, Hullfish et al. (2018a) used fMRI to test the idea that individuals experiencing tinnitus have altered connectivity between the pre-frontal cortex and sub-cortical structures associated with the mesolimbic pathway, specifically the nucleus accumbens. Chronic tinnitus patients were compared with a similar number of healthy controls. Those with tinnitus exhibited distinct patterns not present in controls, whereby significantly increased connectivity was seen even after accounting for the effects of possible confounds like age (tinnitus patients were typically middle-aged adults while controls were young adults) (Hullfish et al. 2018a). TCD appears to be able to explain a relatively divergent set of neurological disorders including examples analogous to tinnitus, particularly neuropathic pain (i.e. pain without an external source) but also Parkinson’s disease and depression (Vanneste et al. 2018).

Attention plays an important role in promoting and modulating tinnitus perception and distress. A scoping review found that attention was consistently impaired in individuals with tinnitus (Vasudevan et al. 2021). The ability of the individual to focus on tinnitus and pull it from competing activity may be expressed as higher network efficiency (Yoo et al. 2021). In fMRI recordings, tinnitus distress is strongly correlated with connectivity between the right executive control network and auditory, default mode, salience and left executive control networks, suggesting tinnitus is a condition of overactive attention (Kandeepan et al. 2019).

The existence of multiple models and mechanisms, with evidence that varies to some degree between studies is a reflection of tinnitus’ heterogeneity. Genotyping may assist in identifying persons at risk for distressing tinnitus. Brain-derived neurotrophic factor (BDNF) (rs6265) may, potentially, be an indicator of susceptibility for tinnitus distress (Vanneste et al. 2021), and the catecholo-methyl-transferase (COMT) polymorphism may be associated with tinnitus loudness (Vanneste et al. 2018).

### Tinnitus assessment and prognosis

Twenty articles between 2007–2023 were reviewed (Figure 2E). There is currently no objective measure that can identify a person as experiencing tinnitus. Multiple forms of assessment can aid in diagnosis and inform research investigating tinnitus mechanisms. There have been attempts to identify subtypes of tinnitus that may be more responsive to certain treatments; however, tinnitus may be best represented as a dimensional disorder varying across a spectrum with indistinct borders between categories (Mohan et al. 2022). Three dimensions of assessment have been proposed to inform clinical practice: 1. Questionnaires (tinnitus severity); 2. Psychoacoustic matching (perception) and; 3. Diagnostic assessment (physiology) (Searchfield and Jerram 2010). Different outcomes from these measures may characterise the role of neural networks responsible for tinnitus (Searchfield and Jerram 2010; Mohan et al. 2022).

Clinical evaluations and research rely heavily on questionnaires and rating scales. The first Tinnitus Research Initiative conference produced a consensus document that provided a framework of recommendations for assessment and ranked them as: essential, highly recommended and might be of interest; it also produced a ‘Tinnitus Sample Case History Questionnaire’ that has been commonly adapted into practice (Langguth et al. 2007). Many tinnitus questionnaires that examine the effects of tinnitus on daily activities have been developed (Langguth, Searchfield, et al. 2011). There has not been universal acceptance of a single index of tinnitus and most were not developed to evaluate treatment outcomes (Langguth et al. 2007). The desirability of a ‘universal’ outcome measure sensitive to treatment effects led to the development of the Tinnitus Functional Index (TFI, Meikle et al. 2012; Henry et al. 2016). The TFI is a 25-item questionnaire with eight subscales (intrusive, control, cognitive, sleep, auditory, relaxation, quality, emotional) and was developed based on USA data. The validity and interpretation of questionnaires can be impacted by translation and target population. In NZ, an evaluation (*n* = 257) of three widely used questionnaires at the time, the Tinnitus Handicap Questionnaire (THQ), the Tinnitus Handicap Inventory (THI) and the Tinnitus Severity Index (TSI), found that reliability and internal consistency were good, but that the THI had a factor structure that differed significantly from the original, meaning direct comparisons between NZ and USA data might be misleading (Searchfield and Jerram 2010). A similar study was undertaken to validate the TFI for NZ. A confirmatory factor analysis and evaluation of internal consistency reliability (*n* = 318) confirmed the same structure in NZ as the USA patients with the primary complaint of tinnitus. In a separate sample (*n* = 40), test–retest reliability, convergent validity and divergent validity were evaluated and found to reflect intended use (Chandra et al. 2018). However, both samples consisted of predominantly older Caucasian males; numbers of Māori and Pasifika were not representative of the NZ population (Chandra et al. 2018).

Tinnitus questionnaires assess multiple dimensions and cannot be considered independent of co-morbidities such as anxiety, depression and hearing loss; for example an evaluation of (*n* = 79) clinical patients identified that elevated low-frequency thresholds were correlated with greater tinnitus handicap (THQ, Searchfield, Jerram, et al. 2007). Quantitative questionnaires, such as the TFI, THQ and THI, are also limited to asking questions that address the majority of tinnitus problems. An individual may have niche tinnitus complaints and unique treatment goals. A semi-qualitative approach for tinnitus based on a goal-setting tool may better address individuals’ specific tinnitus needs. The Client Oriented Scale of Improvement in Tinnitus (COSIT) was developed to enable goal-setting and evaluation of progress towards these goals (Searchfield 2019). A thematic correlational analysis (*n* = 122) comparing the COSIT to THQ, THI and TFI found that individuals differed in their complaints and priorities for treatment, but the most common treatment goals were: 1. reducing tinnitus’ effects on hearing; 2. improved wellbeing; 3. coping with or controlling tinnitus; 4. managing the environmental context; 5. improving sleep; 6. understanding tinnitus. The COSIT measured similar constructs, without strong convergence to the THQ, THI and TFI, indicating that the COSIT could complement the questionnaires and provide an alternative method of assessment (Searchfield 2019).

Aspects of the tinnitus experience can also be captured by psychoacoustic matching of external sound characteristics (pitch, loudness, maskability) to the individual tinnitus (Vajsakovic et al. 2021). The pitch of tinnitus has been of interest mechanistically, as it has an association with hearing thresholds. In a clinical sample (*n* = 192), tinnitus pitch was not correlated with otoacoustic emissions, minimum masking level, edge of hearing loss or worst threshold. Tinnitus pitch was weakly correlated with the frequency at which threshold was approximately 50 dB HL. It was postulated that this may be due to a change from primarily outer hair cell damage to lesions including inner hair cells at these levels of hearing loss (Shekhawat et al. 2014b). Another frequently overlooked feature of tinnitus is its spatial quality. Software implementing psychoacoustic spatial cues can be used to match tinnitus in the horizontal and vertical planes (*n* = 34) with comparable test–retest reliability to pitch and loudness matching (Searchfield et al. 2015). Tinnitus can also affect cognition. An assessment of attention ability in a group of persons with tinnitus (*n* = 16) compared to a matched control group (*n* = 14) found poorer focussed attention for those with tinnitus (Wise et al. 2010).

Although objective measures such as electroencephalography (EEG) have limited ability to distinguish between persons with and without tinnitus, they may play an important role in the prognosis of tinnitus treatment outcomes. A promising approach for prognosis involves developing computational models to predict individual responses to specific tinnitus treatment interventions. Han et al. (2020) investigated the predictive power of pre-treatment cortical oscillatory activity. Their findings suggest that specific patterns of EEG in individuals with tinnitus could predict how well they respond to hearing aid treatment (Han et al. 2020). Clinical improvements with Tinnitus Retraining Therapy (TRT), which combines counselling with hearing aids or low-level neutral sound therapy, have been correlated with pre-treatment EEG measures of the bilateral anterior cingulate cortices that control parasympathetic activity (Kim et al. 2016). Preoperative EEG can also be used to predict tinnitus reduction post-cochlear implants (Song, Punte, et al. 2013). Sanders et al. (2021) applied a spiking neural network (SNN) model to tinnitus-related EEG data to predict individual responses (*n* = 10) to Acoustic Residual Inhibition (ARI) (Sanders et al. 2021). Han et al. (2020) investigated the predictive power of pre-treatment cortical oscillatory activity. Their findings suggest that specific oscillation patterns in individuals with tinnitus could predict how well they respond to hearing-aid treatment (Han et al. 2020). Durai et al. (2021) used a similar model to predict tinnitus-masking benefit in a case series (*N* = 11). The results demonstrated high prediction accuracy (93%–100%), enabling real-time detection and prediction of brain responses to therapies. Doborjeh et al. (2023) explored the potential of combining EEG sensor data with the Tinnitus Functional Index (TFI) score using deep-learning algorithms in eight participants to predict treatment outcomes with 98%–100% accuracy (Doborjeh et al. 2023). Neural networks involved in phantom sound perception are behaviourally segregated but spatiotemporally overlapping (Mohan et al. 2017). Linear support vector machines can accurately differentiate healthy controls from tinnitus patients and low- and high-distress tinnitus (Piarulli et al. 2022). This knowledge could inform the development of targeted therapies aimed at disrupting specific network activity responsible for the phantom sound experience. Pre-treatment objective physiological assessments may unlock personalised management strategies tailored to individual brain dynamics, maximising treatment success.

### Pharmacotherapy

Fourteen articles between 1978–2021 were reviewed (Figure 2F). There is a general consensus that a single drug for treating all forms of tinnitus is unlikely due to heterogeneity in cause and comorbidities (Palumbo et al. 2015). Future drug candidates will be found as we gain greater understanding of the neural basis of tinnitus (Smith et al. 2010). This discovery may be through identification of target medication pathways (Elgoyhen et al. 2014).

The foundation of drug knowledge for tinnitus has been both the result of planning (e.g. animal testing) (Darlington and Smith 2007) and of serendipity (Smith and Darlington 2005). The serendipitous observation in an Auckland pain clinic that intravenous administration of the anaesthetic lignocaine could acutely suppress or eliminate tinnitus engendered significant international interest. In an open study amongst a group of 55 participants with sensorineural hearing loss, 65% had good or excellent tinnitus relief (Meldjng et al. 1978). The ‘lignocaine test’ became a means to probe whether tinnitus was due to central hyperactivity following peripheral deafferentation (Goodey 1981). Following classification according to the lignocaine test, 125 participants were administered the oral anticonvulsants carbamazepine or diphenylhydantoin. Carbamazepine was effective in suppressing tinnitus in patients (56%) who had a good or excellent response to intravenous lignocaine. Patients who had little or no response to the lignocaine test did not respond to oral anticonvulsants. Sodium valproate (an anticonvulsant that blocks voltage-dependent sodium channels and increases the levels of GABA) has been reported to be effective in case reports (Goodey 1981; Menkes and Larson 1998). A limiting factor in use of anticonvulsants is their side-effect profile (Goodey 1981).

Potential candidate drugs have been generated through tinnitus patients reports of tinnitus being reduced or acutely eliminated following recreational drug use. Cannabinoid receptors in tinnitus and the potential of Cannabis and cannabinoid agonists has been the focus of several reviews (Smith 2011; Smith and Zheng 2016; Zheng and Smith 2019). The psychoactive drug 3,4-Methylenedioxymethamphetamine (MDMA) was explored compared to placebo at a dose of 30 mg in five participants behaviourally and at a dose of 70 mg behaviourally and with fMRI (Searchfield, Poppe, et al. 2020). Behavioural effects were no greater than placebo, but the fMRI indicated the right post-central gyrus and right posterior and superior temporal gyrus and the thalamus and frontoparietal network had greater connectivity post-MDMA. Natural remedies for tinnitus have also been explored. *Ginkgo biloba* extract has been advertised as a tinnitus treatment; a review found that two well-conducted double-blind placebo controlled studies indicate efficacy at the level of placebo (Smith et al. 2005).

Glutamate antagonists administered to the cochlea may prevent the centralisation of tinnitus by blocking aberrant afferent activity acutely post-injury. A Phase1/2 double-blind, placebo-controlled trial of a single intratympanic injection of OTO-313 resulted in a clinically meaningful reduction of tinnitus in 43% of participants compared to 13% in the placebo group; the drug was well tolerated and was recommended for advance to larger trials^1^ (Maxwell et al. 2021).

### Neuromodulation

Twenty-eight articles between 2010–2022 were reviewed (Figure 2G). Neuromodulation is the application of electrical or magnetic stimuli to modify nervous system activity. A large variety of different neuromodulatory interventions have been developed and are adapted to new insights in the pathophysiology of tinnitus. rTMS targeting the temporal, temporoparietal and frontal cortex have been the mainstay of non-invasive neuromodulation. Evidence to support the clinical implementation of rTMS for tinnitus is limited (Stinear 2010; Langguth et al. 2015). In NZ, electrical neuromodulation approaches have been investigated, rather than rTMS.

Intracranial auditory cortex stimulation via implanted electrodes has been trialled to treat severe cases of intractable tinnitus. De Ridder has pioneered neurosurgical direct stimulation procedures through longstanding collaborations in Europe. Two patients with severely distressing tinnitus had implantation of electrodes on the Dorsal Anterior Cingulate Cortex; one responded, one did not. The responder had increased functional connectivity between the parahippocampus, the subgenual anterior cingulate cortex and the insula, while the non-responder had decreased connectivity (De Ridder et al. 2016). Resting-state functional connectivity differed in five patients who responded and five patients who did not respond to auditory cortex implantation. Activity between the auditory cortex and parahippocampus may predict response to cortical implants (De Ridder and Vanneste 2014b). In another case, complete resolution of pure-tone tinnitus but not noise-like tinnitus was obtained. Subsequent insertion of a subcutaneous electrode for C2 nerve stimulation improved the noise-like tinnitus by 50% (De Ridder and Vanneste 2015).

An alternative to direct stimulation of the brain is stimulation through the scalp and skull. Various types of low-level electrical current are passed between cathode (positive) and anode (negative) electrodes placed on the head overlaying target stimulation areas. Anodal tDCS of the left temporoparietal area was carried out with two current intensities (1 mA and 2 mA) and three durations (10 minutes, 15 minutes and 20 minutes). A current intensity of 2 mA for 20 minutes was the more effective stimulus parameter for tinnitus suppression (Shekhawat, Stinear, et al. 2013). In a similar study, tDCS of the dorsolateral prefrontal cortex (DLPFC) found no significant difference between intensity and duration of stimulation. As the number of sessions increased, there was a greater reduction in the tinnitus loudness that plateaued after six sessions (Shekhawat and Vanneste 2018b). Anodal transcranial direct current stimulation (tDCS) over the left auditory cortex was found to be more effective in reducing tinnitus annoyance than cathodal stimulation (Joos et al. 2014). There was no difference in the results for tinnitus loudness and the distress experienced when the anode was placed on the right dorsolateral prefrontal cortex and the cathode was on the left DLPFC or the shoulder (Rabau et al. 2017). A meta-analysis of tDCS effectiveness in tinnitus management found few studies, but all demonstrated significant tinnitus intensity improvement (Song et al. 2012), however, it is still to be determined if tDCS is clinically beneficial (Shekhawat, Stinear, et al. 2015; Lefaucheur et al. 2017).

Alternative electrical stimulation types are transcranial alternating current stimulation (tACS) and transcranial random noise stimulation (tRNS). tRNS can be sub-categorised as low-frequency tRNS (lf-tRNS), high-frequency tRNS (hf-tRNS) and whole-frequency spectrum tRNS (wf-tRNS). A comparison of tDCS, tACS and tRNS found that tRNS induced the larger transient suppressive effect (Vanneste, Fregni, et al. 2013). Bifrontal tDCS stimulation (anode and cathode over DLPFC) modulated tinnitus annoyance and tinnitus loudness, whereas tACS did not (Vanneste, Walsh, et al. 2013). In another study, a single session of tRNS induced a significant suppressive effect on tinnitus loudness and distress, whereas tACS did not. Multiple sessions of tRNS resulted in greater tinnitus loudness suppression without tinnitus distress decreasing (Claes et al. 2014). hf-tRNS and lf-tRNS significantly reduced tinnitus loudness, lf-tRNS reduced tinnitus distress and hf-tRNS had a greater effect of loudness and distress in pure-tone tinnitus compared to narrow-band noise tinnitus (Joos et al. 2015).

Conventional tDCS is modelled as stimulating a broad region of the brain. High-definition tDCS (HD tDCS) uses smaller electrodes to limit current spread. Many of the HD studies mirror those previously undertaken for tDCS. HD-tDCS of the LTA and DLPFC were compared (*N* = 27) across two sessions with four different settings (1 mA, 10 minutes; 1 mA, 20 minutes; 2 mA, 10 minutes; and 2 mA, 20 minutes). The stimulation of the LTA and DLPFC were equally effective, with a current intensity of 2 mA for 20 minutes providing greatest tinnitus relief (Shekhawat et al. 2016). In a double-blind, sham-controlled, randomised trial (*N* = 13) of HD-tDCS to the DLPFC, stimulation resulted in transient tinnitus loudness suppression after 15 minutes (Shekhawat and Vanneste 2018a). However, in another study, a tDCS group and the HD tDCS group showed no differences over time (Jacquemin et al. 2018). As with low-definition electrical stimulation, alternatives to direct current have been explored. For example HD-tIPNS is safe, but no behavioural benefit was found despite EEG changes (Smeele et al. 2023).

Further stimulation options include multisite electrical stimulation and bimodal stimulation (De Ridder and Vanneste 2014a; To et al. 2018; De Ridder, Adhia, et al. 2021). A multisite treatment protocol of bifrontal tDCS before bilateral auditory cortex tRNS resulted in greater effects than bifrontal tDCS alone (To et al. 2017). Loud auditory stimulation before, during and after tDCs was compared in nine participants, of whom seven reported transient tinnitus suppression when sound stimulation was present during tDCS (Shekhawat, Kobayashi, et al. 2015). The bi-modal approach includes vagus nerve stimulation with an implanted device paired with tonal auditory stimulation. In a case study, this stimulation resulted in a significant reduction in tinnitus symptoms that lasted for two months (De Ridder, Kilgard, et al. 2015). In a small trial, 40% of participants exhibited clinically meaningful improvements in Tinnitus Handicap Inventory scores and reductions in minimum masking level that lasted at least two months (De Ridder, Vanneste, Engineer, et al. 2014). Direct vagal nerve stimulation paired with tones results in significant, moderate reductions in tinnitus distress and loudness (De Ridder, Langguth, et al. 2021).

### Sensory therapies

Thirty-six articles between 2007–2023 were reviewed (Figure 2H). Sounds, and to a limited degree visual and tactile stimuli, have been used in tinnitus therapy to reduce tinnitus perception and its effects. Sensory-based therapies for tinnitus have been covered extensively in NZ research and NZ-authored reviews categorising the therapy (Searchfield, Cameron, et al. 2010; Hoare et al. 2014; Searchfield et al. 2017; Searchfield et al. 2019a; Searchfield 2021).

Sound has, understandably, been the primary mode of sensory stimulation. The first wearable technology for sound therapy was the hearing aid. A scoping review identified 17 studies of which all but 1 concluded hearing aids were beneficial in managing tinnitus (Shekhawat et al. 2013). There are many potential mechanisms of benefit, such as improved hearing, less social deprivation, enriching auditory input, reducing attention on tinnitus and tinnitus masking through amplification of background sounds (Searchfield et al. 2019a). Hearing aids with psychoeducation (*n* = 29) provided benefit above psychoeducation alone (*n* = 29) (Searchfield, Kaur, et al. 2010). Stimulation across a wide frequency range is considered important (Shekhawat, Searchfield, Kobayashi, et al. 2013) but frequency lowering of high-frequency speech sounds so they are audible (*N* = 16) does not reduce tinnitus further than normal amplification (Hodgson et al. 2017).

Hearing aids are most effective long term when tinnitus pitch is within their amplification range and is masked when the hearing aids are first fitted (McNeill et al. 2012). The implication is that tinnitus masking is the primary beneficial mechanism through which hearing aids act on tinnitus. Masking reduces the audibility of tinnitus by covering (complete masking) or incomplete covering (partial masking) with sound (Searchfield 2021). The masking response is heterogeneous and appears to be the result of informational (central) masking, as demonstrated by the ability of sound contralateral to tinnitus being able to mask (*n* = 8) (Kemp and George 1992b) and unpredictable sounds resulting in lower tinnitus annoyance and loudness after acute administration than predictable sounds (*n* = 23) (Durai, Kobayashi, et al. 2018b).

Masking through amplification of background sounds using hearing aids becomes increasingly difficult with hearing loss of low-frequency sounds McNeill et al. (2012). In the presence of low-frequency hearing loss, the use of sounds generated by the hearing aids, or streamed via Bluetooth, are recommended in combination with amplification (Carrabba et al. 2010). The sounds used alongside the amplification are typically broad band noise (BBN) and nature sounds. In a multi-country trial (Italy and NZ) clinical outcomes appear independent of allocation to BBN (*n* = 17) or nature sounds (*n* = 20) (Barozzi et al. 2016). An attempt to augment hearing aid use through priming the hearing system with tDCS did not enhance hearing aid outcomes (*n* = 20) over hearing aids and sham-tDCS (Shekhawat et al. 2014a). New Zealand clinical experiences in using hearing aids for tinnitus have been widely disseminated internationally as clinical protocols (Searchfield 2008; Searchfield 2015b).

For some listeners, a post-masking ARI can occur. ARI refers to a temporary reduction in tinnitus perception after a period of loud sound. Personalising ARI stimuli by matching them to individuals’ tinnitus pitch increased likelihood and length of the RI effect (Sockalingam et al. 2007). ARI has more recently been used to examine neurophysiological biomarkers of tinnitus (King et al. 2021), where EEG (*N* = 30) showed increases in power spectral density of alpha and gamma bands related to ARI periods compared to control conditions (stimulus presented at threshold).

Aside from masking, sound may also act to divert attention from tinnitus and reduce negative reactions (Searchfield 2021). Sound is used in Tinnitus Retraining Therapy to putatively facilitate habituation. Differences in EEG before to six months after TRT are consistent with reduced top-down autonomic responses (Lee et al. 2019). Some sounds may affect tinnitus distress more than perception, for example, nature sounds and music are thought to promote relaxation, reducing negative affective response to the tinnitus. Music is effective in the short-term management of tinnitus (*n* = 13) but the music should be selected by the listener (Hann et al. 2008). The parameters of surf/ocean wave sounds (*n* = 10) and simulated surf sounds (*n* = 10) were found to have varying responses from participants. There was a preference for slow oscillating damped sounds over fast ramped sounds (Searchfield 2019b). Addition of binaural beats at 8 Hz to an ocean sound was not found to improve tinnitus (*n* = 20) above the ocean sound alone (Munro and Searchfield 2019). More complex sound therapy approaches include an attempt to recategorise tinnitus perception from an unnatural (phantom) perception to a naturally occurring real-world sound object (e.g. bird song) that led to a significant reduction in the TFI (Durai et al. 2021). Despite continued interest in novel sounds, a cross-over trial in 18 participants found outcomes to nature sounds and BBN to be equivalent (Durai and Searchfield 2017). Rating measures of tinnitus (*n* = 21) improved after 30 minutes of stimulation with either BBN or nature sounds, but only BBN reduced objective blood pressure measures (Aydin and Searchfield 2019). An alternative method to improve tinnitus masking is to manipulate spatial attributes of sound. Perceived masker location can be modified to appear from the same position as tinnitus (in ears/head). Across three studies, participants (total *n* = 34) had a preference for the spatial masking (Searchfield et al. 2016).

Most sound therapies are passive and do not require directed listening effort to prescribed tasks. Perceptual training paradigms require active effortful listening and aim to ‘rewire the brain’ by strengthening neural pathways that counter the maladaptive plasticity and abnormal activity associated with tinnitus. Induction of plasticity requires repetition of (adaptive) processes. Tasks may be fundamental psychoacoustic tasks such as categorisation or discrimination (Jepsen et al. 2010) or require schema-level tasks identifying and localising auditory objects in competition with other sounds (Searchfield, Morrison-Low, et al. 2007). Using frequency and categorisation tasks (*N* = 20), Jepsen et al. (2010) found a small effect on THI scores with the discrimination task and ability to ignore tinnitus with categorisation training. Searchfield et al. (2007) found that performing a selective attention task that increased in difficulty over 15 days reduced tinnitus loudness matches in 6 of 10 participants and MML in 8 of 10 participants. Participant engagement and motivation has been successfully achieved through ‘gamification’ of auditory training paradigms. In a task involving the spatial location of a target sound while including distractors, including a tinnitus Avatar, a feasibility study (*n* = 8) found that training for 30 minutes per day, for 20 consecutive days, resulted in significant reduction in THI (Wise et al. 2015). In a small RCT, 15 participants who played the attention-training game had significant changes in TFI scores, while 16 participants who played a control game (a visual-only version of the falling-block game ‘Tetris’) improved less. Changes in TFI scores were correlated with changes in attention tasks and Auditory Evoked Potentials (Wise et al. 2016). To be highly effective, sound-based therapies may need to incorporate more than one strategy. A randomised controlled trial of a digital ‘polytherapeutic’ developed in NZ with both effortless and effortful strategies demonstrated clinically meaningful changes in tinnitus over 12 weeks (*n* = 31), which an equivalent effortless-only therapy (*n* = 30) did not (Searchfield and Sanders 2022).

One of the benefits of perceptual training is that other sensory modalities such as vision and tactile sensation can be combined with hearing to leverage multisensory integration processes. Multisensory perceptual training combining auditory, visual and tactile stimuli were shown to reduce tinnitus severity and symptom ratings (*n* = 18) (Spiegel et al. 2015) and induce connectivity changes in sensory and attentional neural networks (*n* = 20) (Searchfield, Spiegel, et al. 2021). A virtual reality system has been tested (in 18 healthy participants) that aims to utilise these concepts and deliver them in an immersive manner (Draper et al. 2023). Future sound therapies are likely to incorporate extensive use of biosensory measures along with machine learning to modify sounds and activities to adapt and personalise as therapy progresses (Searchfield, Sanders, et al. 2021).

### Clinical practice

Twenty-three articles between 2007–2020 were reviewed (Figure 2I). The majority of the articles were narrative reviews. The value of clinical guidelines in the absence of a single universally accepted, gold-standard, treatment has been debated. It has been argued that concrete clinical guidance including use of evidence-informed expert opinion is important (Searchfield 2011) when there is not the rigour in research to enable solely evidence-based practice (De Ridder, Vanneste, Elgoyhen, et al. 2015). Such guidance is particularly needed because there are a plethora of alternative therapies and gadgets marketed to clinicians and directly to patients. An example of a direct-to-consumer tinnitus device was the Reltus ear massager, purported to reduce tinnitus by vibration. A study (*n* = 23) undertaken to evaluate its short-term effectiveness and mode of effect found that the likely mechanism of effect was through the sound it made, rather than tactile stimulation (Jonsson et al. 2016).

The heterogeneity of tinnitus needs to be accounted for in research methodology and contributes to the challenge that clinicians face in managing tinnitus (Landgrebe et al. 2012; Cederroth et al. 2019). New Zealand researchers have played significant roles in the development of a widely used algorithm for the diagnostic and therapeutic management of tinnitus (Langguth, Biesinger, et al. 2011). This model provides a flow chart of patient presentation through diagnosis to broad therapy recommendations. This approach has been built upon the experiences of clinicians who have provided overviews of their clinical approaches in review articles (Goodey 2007, 2010a, 2010b; Langguth, Kreuzer, et al. 2013). Common aspects to practice include the correction of any underlying cause (e.g. ear wax, conductive hearing loss), addressing aggravating factors (e.g. stress, diet, side effects of medications, head-jaw-neck injuries and manipulation) (Enrico and Goodey 2011), providing psychological therapies (e.g. counselling, cognitive behavioural therapy) and sensory enrichment (e.g. hearing aids, sound therapy) (Searchfield 2015a). A single 60–90 minute session of tinnitus counselling (*n* = 16) can be enough to result in a clinically meaningful reduction in 50% of patients (Searchfield, Boone, et al. 2020). Comprehensive descriptions of counselling and psychoeducation for tinnitus, based on NZ practice, have been published (Searchfield et al. 2011).

Although the core principles of good clinical practice in tinnitus have not dramatically changed in the last 75 years (Searchfield 2015a), there is potential for new diagnostic and treatment methods (Moller et al. 2015). Personalised approaches potentially applying polytherapeutic methods may be necessary (Searchfield, Zhang, et al. 2021). However, new therapies will only be beneficial if they can be accessed. A survey predominately of Australians with tinnitus identified five themes for improved service delivery: 1. Tinnitus alleviation; 2. Government policies for tinnitus; 3. Reduced barriers; 4. Self/public awareness; 5. Hearing devices (Mui et al. 2023). Internet-based therapies may be solutions to improving treatment access, another is to ensure that material is understood. Internet-based cognitive behaviour therapy is a relatively easily accessed therapy; in a German sample, 73% showed meaningful improvement (Probst et al. 2019). It is important that information on the Internet is readable; a review of 134 websites identified great variability in their quality and useability for people with low literacy (Manchaiah et al. 2019). A study comparing a tinnitus brochure (*n* = 12) to a version optimised for readability (*n* = 12) found that the group provided with the revised version had greater self-efficacy and comprehension of the material (Ming and Kelly-Campbell 2018).

Tinnitus care is best provided in a multidisciplinary environment (Shakes 2010). In NZ, Audiology has been the primary profession for tinnitus rehabilitation. Although audiologists should be capable of offering comprehensive tinnitus services (Searchfield and Baguley 2011), there is limited time devoted to tinnitus in clinical training programs. A survey (*N* = 25) of a continuing education (CE) workshop for NZ audiologists identified the need for additional training and reported benefits of CE, but participants also wanted ongoing support and mentoring (Searchfield, Fok, et al. 2020). Audiologists report counselling as an area of practice in which they would like to be more confident.

## Discussion

**Figure 4.**
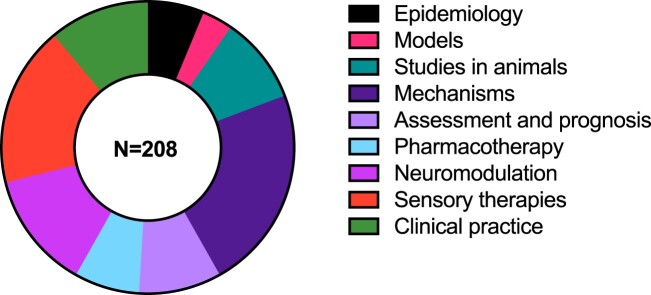
Proportion of articles per theme for the review.

Nine themes were identified from 208 publications from 1978–May 2023: 1. Epidemiology; 2. Models; 3 Mechanisms; 4. Studies in animals; 5. Assessment and prognosis; 6. Pharmacotherapy; 7. Neuromodulation; 8. Sensory therapies; 9. Clinical practice. Publications could have been allocated to multiple themes but were allocated only to one. The fewest publications were on the theme ‘models’, the most on ‘mechanisms’, while the other themes contained similar numbers of publications (Figure 4). All research methodologies including review, qualitative, case-based and quantitative methods were included, indicating an inclusive and broad review. Reflecting international trends, the number of tinnitus publications with NZ authors has risen sharply in the last two decades.

Most outputs came from the Universities of Otago and Auckland, with significant contributions from the University of Canterbury, AUT university and, especially in early research, public hospitals. Most of the animal studies and research on tinnitus mechanisms in humans were affiliated with the University of Otago. Most sensory therapy research was from the University of Auckland. Other research interest areas were shared across institutions. The results are consistent with international reviews of contributions to tinnitus that identify these centres as important contributors to international research (Zhou et al. 2022; Ye et al. 2023).

A strength of NZ tinnitus research is both its breadth and depth. The nine themes cover a wide range of tinnitus topics, and within each topic many methods are used. The breadth does not appear to have been at the sacrifice of depth of knowledge as areas of strength have emerged over time. Tinnitus research in NZ began in the 1970s with pioneering work using intravenous lignocaine (Meldjng et al. 1978). Using animal models of tinnitus, NZ researchers have made significant contributions to the understanding of the pathophysiology and treatments of tinnitus (Zheng and Smith 2019). There has been significant impact, such as through widespread adoption of questionnaires developed with the input of NZ researchers (TFI (Meikle et al. 2012), COSIT (Searchfield 2019)). NZ researchers have spearheaded research into electrical stimulation (De Ridder, Adhia, et al. 2021) and sound stimulation (Searchfield et al. 2017) for tinnitus. Recently, bimodal brain stimulation approaches (De Ridder, Langguth, et al. 2021) and digital therapies have revealed promising results (Searchfield and Sanders 2022). It appears that targeting different sensory modalities or sensory and psychological domains are more promising than single-target approaches. A new, emerging, strength in NZ tinnitus research is the use of artificial intelligence/machine learning for treatment prognosis and, in the future, real-time selection of personalised therapies (Doborjeh et al. 2023). This approach has the potential to detect patterns in subjective reports and individual variability in brain activity, leading to identification of therapy targets, potentially in real time (Searchfield, Sanders, et al. 2021). A strong theoretical and pathophysiological approach from NZ researchers has led to important contributions in both developing a widely accepted definition for tinnitus and tinnitus disorder (De Ridder, Schlee, et al. 2021), as well as involvement in the development of international treatment (Lefaucheur et al. 2017; Langguth et al. 2023) and research guidelines (Landgrebe et al. 2012). A feature of much of the research has been collaboration, particularly with countries in the European Union.

One of the fundamental questions addressed by much of the research is the understanding of neurological dysfunction and tinnitus. There is ongoing interest in unravelling details such as the locus of dysfunction, associated functional brain networks in auditory and non-auditory areas, alterations thereof and causality (De Ridder et al. 2022). In addition to the processes leading to a tinnitus sensation, NZ researchers have contributed answers to the question of why tinnitus can change with environmental factors, such as emotion (Searchfield 2014). Some individuals seem to suffer from their tinnitus more than others, e.g. experience stress, anxiety or decreased cognitive performance. Individual differences in personality play a strong role (Dawes and Welch 2010). Investigating brain networks has become a primary tool for quantifying biomarkers and probing tinnitus-generating mechanisms in humans (De Ridder and Vanneste 2021). The method of choice in NZ to explore brain functioning has been EEG, and there is expertise in this tool across the nation’s tinnitus research groups. The use of artificial intelligence as a prognosis tool is an emerging area of expertise (Doborjeh et al. 2023). New Zealand research has shown that there are different tinnitus-generating mechanisms, based on a range of factors such as the extent and pattern of hearing loss, speed of onset, age and experience, cognition and comorbidities. This research has been facilitated by an open innovation research environment that enables innovative research and rapid clinical translation.

In the scoping review approach the quality of the data is not assessed. For studies that recruited participants in NZ, numbers were recorded, and study numbers ranged from a few participants in psychoacoustic evaluations through to one of the largest population studies of tinnitus prevalence internationally. A number of human studies were feasibility or proof of concept studies and evaluating novel therapies; appropriately some of these have progressed to larger controlled trials. We may not have identified all the relevant studies in the field. The use of other terms or more than the two databases may have yielded additional published scoping reviews. However, given that several of the authors have been active in this field for 20 years or more, and are based in NZ, we are confident that few published pieces of research were omitted. However, the tendency to be able to publish positive results may mean that null or negative findings are not fully represented.

There are clear gaps in our understanding of the impact of tinnitus on Māori and minority communities in NZ. In stark relief to the volume of tinnitus research published is the absence of consideration of ti nga taringa amongst Māori. We know tinnitus is as high amongst older Māori as it is among older NZers of European descent (Wu et al. 2015) but beyond this we know very little. The review indicated that traditional knowledge and beliefs have not been considered in published tinnitus research to date. This gap must urgently be addressed. Effort is needed to reduce inequality and improve the translation of research to good tinnitus treatment outcomes for all our communities. Larger trials should strive for equal explanatory power with deliberate recruitment of Māori, and there should be specific investment to encourage Māori researchers into the tinnitus field.

A further weakness is that, despite NZ being a world leader in tinnitus research, there are few clinical services focussed on tinnitus, and these are in larger centres. The review has identified strengths in audiological and oto-neurological clinical practice, whereas psychology-based research is less evident and presents an opportunity. Tinnitus is not a priority for publicly funded hospitals, so the possibility of a government-funded tinnitus service, such as in the United Kingdom (McFerran et al. 2018) seems unlikely. But researchers, clinicians and the public can play a role in educating government funders such as NZ’s universal no-fault accident insurer (Accident Compensation Corporation) and Veterans affairs that tinnitus is a disorder in its own right, not just a symptom of hearing loss. The community also has a role in lobbying government to address the absence of subsidies for tinnitus care equivalent to the support provided for hearing loss and encouraging private health insurers to cover tinnitus disorder related claims. New Zealand researchers should take a bigger role in providing the community with the evidence needed to support calls for improved service delivery and funding.

It is quite remarkable what has been achieved in tinnitus research given NZ’s small population, number of researchers and competitive funding environment. New Zealand is ranked in the top 10 countries internationally for tinnitus expertise (before any per capita correction or consideration of research expenditure) (https://expertscape.com/ex/tinnitus). Continued investment in this world-class research is essential. An effective suite of treatments could lower costs (estimated to be over NZ$ 3.5 billion, Deloitte Access Economics 2017) to the NZ economy by reducing lost productivity and service delivery costs. Historically the Deafness Research Foundation had a limited amount of annual funding across hearing sciences, though recently the Eisdell Moore Centre has allocated some of that funding to tinnitus. The Auckland Medical Research Foundation has managed the JM Cathie trust, the only NZ fund dedicated to tinnitus research. The Tinnitus Research Initiative (TRI) was founded by the philanthropist Dr M De Nora. New Zealand researchers have benefited through collaborating with TRI and Dr De Nora’s direct support of NZ research. New Zealand researchers have also received international research grants and collaborated with international industry partners. It is hoped that in the future there are funding opportunities to sustain tinnitus research and enable emerging researchers in this space to flourish. The review has identified the abilities of NZ tinnitus researchers to undertake cutting-edge research. These findings should help NZ tinnitus researchers create cases for funding, particularly amongst international funders who may be unaware of NZ’s research capabilities. The success of NZ researchers has, in some part, been due to serendipity. The authors believe other contributing factors to our success have been an environment geared towards innovation, collegiality, collaboration and having open minds. The ‘Tyranny of Distance’ to other research groups has, perhaps, built self-reliance and an ability to ‘make-do’ with resources. On the other hand, the ability, and importance, to collaborate internationally is evidenced by the high number of co-authored papers, especially from Europe. Globally researchers may see if there are lessons to be learnt from the growth of tinnitus research in NZ.

## Conclusion

New Zealand researchers have played a significant role in advancing the science and clinical practice of tinnitus. New Zealand has been identified as a world leader with significant impact in the field of tinnitus. The review identified strengths in research of epidemiology; models; mechanisms; studies in animals; assessment and prognosis; pharmacotherapy; neuromodulation; sensory therapies; and clinical practice. The research was characterised by being innovative and collaborative. However, despite positive contributions, little is known about tinnitus amongst minority communities and Māori, the tangata whenua, indigenous people of Aotearoa/NZ. This significant shortcoming needs to be addressed.

## Note

1 The subsequent trial published outside of the scoping review time window was negative for benefit.
